# Uncovering mathematics teachers’ instructional anticipations in a digital one-to-one environment: A modified UTAUT study

**DOI:** 10.1016/j.heliyon.2024.e35381

**Published:** 2024-08-03

**Authors:** Robert Weinhandl, Christoph Helm, Branko Anđić, Cornelia S. Große

**Affiliations:** a School of Education, Johannes Kepler University, Department of STEM Education, Altenberger Straße 68 4040 Linz. Standort. *Science Park* 5, Austria; b School of Education, Johannes Kepler University, Department of Education Research, Altenberger Straße 68 4040 Linz. Standort. Science Park 5, Austria; c University of Vienna, Austrian Educational Competence Center for Biology, Porzellangasse 4/2/2 1090 Wien, Vienna, Austria

**Keywords:** Digital one-to-one approach, Mathematics teachers' instructional anticipations, Modified UTAUT, lower secondary education

## Abstract

More and more digital technologies are being integrated into school learning, and one strategy by policymakers to reinforce this trend is employing digital one-to-one approaches. For digital technologies to be fruitfully integrated into school-based learning scenarios, teachers and their anticipations are key. In our study, we want to explore how internal, external and technological factors affect the instructional anticipations of mathematics teachers in a digital one-to-one educational environment. Therefore, we employed a modified model of the Unified Theory of Acceptance and Use of Technology. Through our structural equation study, in which data from more than 900 mathematics teachers were analyzed, we identified that especially technological and external factors can predict mathematics teachers’ instructional anticipations. Findings from our study could be particularly relevant for educational policymakers, informing them about the importance of factors or interventions related to educational technology implementation.

## Highlights

• The research was conducted in a newly initiated one-to-one teaching environment.
• Personal Initiative (PI) is positively associated with Perceived Pedagogical Impact-PPI.
• User Interface Quality (UIQ) positively predicts PPI and PerformanceExpectancy-PE.
• Technology Compatibility significantly predicts PE.
• UIQ has a greater impact on older teachers' perceptions of technology than on younger ones.

## 1 Introduction

In 2023, global spending on educational technologies exceeded USD 140 billion^1^ [1], leading, among other things, to more and more digital one-to-one approaches being implemented in secondary schools in different countries. One-to-one digital approaches in education refer to using one digital device for each student, ensuring student-device interaction and individualization, and enhancing engagement and learning outcomes [2,3]. Such approaches will increasingly characterise teaching and learning in schools in the future.

Since the school year 2021/2022, students at the beginning of secondary school (5th grade, 10-year-old children) have been equipped with digital devices (DD) in Austria [1]. These DD are notebooks or digital tablets with a keyboard and a digital pen. Through this, Austria follows a digital one-to-one approach, meaning all students are provided with their own DD. Uruguay [4] and Peru [5], as well as South America in general, were some of the first and most widespread adopters of digital one-to-one programs in schools [6]. While Norway, Greece, Portugal and Spain are the European pioneers in digital one-to-one approaches [7], the authors emphasise a discrepancy between the potential and recommendations of policy makers and the actual implementation of digital technologies in classrooms. In relation to the teaching and learning of mathematics, Hull and Duch [8] have shown that digital one-to-one approaches had no significant impact on students' learning outcomes in the short term and achieved only slight improvements in student scores in the medium term. In addition, this learning environment means that students need more time to complete their homework when using DD.

Using digital technologies in mathematics classrooms has a long tradition, with early studies dating back to the beginning of digital technologies in education [9]. In line with many experts [[10], [11], [12]], teachers' anticipations and beliefs are key to using technology effectively in mathematics lessons. This study examines the expectations of mathematics teachers about their teaching when both students and teachers use the same DD. This is interesting because some of the challenges of using technology in mathematics class, like unequal access to devices, have been lessened by policymakers. Unlike other studies that looked at how teachers adapt lessons to different digital devices [12], our research focuses on a setting where students and teachers use the same device. Given these circumstances, our research aims to identify how factors like mathematics teachers' personal innovativeness, facilitation conditions or technology compatibility affect mathematics teachers’ instructional anticipations. To achieve this, we are using a modified version of the Unified Theory of Acceptance and Use of Technology (UTAUT), which is another novelty of our study. In this study, we refer to the modified UTAUT in which, in addition to the general UTAUT constructs, constructs were added that relate exclusively to education, such as Perception of Pedagogical Impact (PPI), but also the construct Anxiety (ANX). By figuring out what affects teachers' expectations, this research can provide valuable insights for anyone involved in improving mathematics education with technology, such as teacher trainers, school leaders, educational policymakers and other educational stakeholders.

To help readers understand our research better, we'll first explain the specifics of teaching mathematics in a digital environment and then elaborate on findings regarding UTAUT research in teaching mathematics contexts.

### 1.1 Teaching mathematics using technologies

In terms of student achievements and using technologies, Wenglinsky [13] noted 25 years ago that the relationship between the use of educational technology and student achievement in mathematics is ambiguous, which has been confirmed in the recent past by several studies [14,15]. Among other things, the success of using educational technologies in mathematics lessons depends on the quality of instruction. To provide good technology-enhanced mathematics instruction, mathematics teachers need a comprehensive understanding of content, technology and pedagogy [16].

In addition to being one key to educational success through technology, according to many experts [12,17,18], mathematics teachers represent a fundamentally important factor in the basic implementation of technologies in mathematics lessons. According to McCulloch et al. [12], one of the most critical factors regarding using technologies in educational contexts is the extent to which using technologies can be linked to achieving instructional goals. In this context, technologies for learning mathematics span a wide range, from mathematical action tools to general communication and collaboration tools. Which tools are used in mathematics lessons and which are not is often decided by the ease of use attributed to the tools by teachers [12]. According to Lavicza [11] and Thurm and Barzel [19], mathematics teachers' knowledge, beliefs, and conceptions regarding the use of technologies determine the integration of technologies into mathematics lessons and the pace of this. In line with Misfeldt et al. [20], mathematics teachers tend to have pre-mastery beliefs about technology use. Teachers with pre-mastery beliefs tend to be situated between technology supporters (exploratory beliefs, i.e., teachers who believe that technologies should be used to learn mathematical concepts) and technology sceptics (post-mastery beliefs, i.e., that technologies should only be used when students can solve mathematical problems using a paper-and-pencil approach). Different theories or models have been used recently to determine mathematics teachers’ acceptance of technology use. Well-known theories or models are the Technology Acceptance Model [21] and the Unified Theory of Acceptance and Use of Technology [22]. UTAUT, developed by Venkatesh et al. [23], consisted of four basic factors: performance expectancy, social influence, effort expectancy and facilitating conditions. According to these authors, performance expectancy indicates the extent to which users of a technology believe that it will help them achieve better performance. Effort expectancy indicates the belief of the user on how easily an individual can use a particular technology [23]. Social influence refers to the degree to which others believe it is important for them to use the new technology [23]. The same authors define facilitating conditions as the degree to which users believe that there is organisational and technical support for the use of the new technology.

Our study aims to discover the anticipations of mathematics teachers towards technologies and the use of technologies for teaching and learning mathematics. In order to investigate the anticipations of mathematics teachers towards technologies and the use of technologies for teaching and learning mathematics, we used a modified form of the UTAUT in our study. Many studies [[24], [25], [26]] suggest that UTAUT is valuable for understanding technology use in mathematics education. Research which is based on UTAUT can provide knowledge for sustainable research-based support necessary to realise the full potential of technologies in mathematics classrooms, and the results of UTAUT studies can provide vital information for widening and improving the use of technologies in mathematics lessons.

### 1.2 Unified Theory of Acceptance and Use of Technology in education

Some experts [[27], [28], [29], [30]] consider that the UTAUT model can be a helpful tool for understanding technology adoption and use, but its usage and usefulness may vary depending on the context and region. According to Tappe [31], the UTAUT provides both a theoretical foundation and a methodological framework, and Khechine et al. [27] and Tamilmani et al. [32] found that the UTAUT model is simple to use, accurate and robust in predicting technology adoption and use. Cain [33] argues that using educational research can change teachers’ decisions and that UTAUT can help understand how teachers adopt new technologies and practices. Similarly, UTAUT can also be used to identify barriers and challenges teachers face in adopting digital technologies in their teaching [34].

The study's results by Al-zboon et al. [35] show that science and mathematics teachers' anticipations towards integrating information and communication technology in education processes were high and positive. Teachers had positive perceptions of integrating information and communication technologies in all dimensions (performance expectancy, effort expectancy, social influence, and facilitating conditions), with expected performance having the most impact on teachers' anticipations and facilitating conditions the least. The study by Saal et al. [36] shows that social influence most significantly impacts mathematics teachers' acceptance and use of technology. Furthermore, facilitating conditions such as adequate technological infrastructure and information technology influence teachers' use of educational technology in mathematics classrooms. Also, authors [22] discovered that social influence significantly affects mathematics teachers' behavioural intention to use digital technologies.

In contrast, Stols et al. [37] found that social influence has little impact on teachers' acceptance and use of technologies. Regarding facilitating conditions, teachers had access to adequate equipment and infrastructure (although not always under ideal conditions) but did not have sufficient skills to use it. Furthermore, the study by Stols et al. [37] showed that mathematics teachers’ perceptions of their limited abilities outweighed external facilitating conditions such as sound equipment. Although teachers know the potential value of using technology, they are still hesitant to use it in their teaching. Participants found the use of technology “overwhelming” because of their limited confidence in their technology-related knowledge and skills.

Many studies on the acceptance of digital technologies by mathematics teachers [35,37] show that further development and training in technological skills are needed. In this context, the study by Gurer [21] indicated that such training or professional teacher development programmes are also relevant for prospective and novice teachers, and according to Hero [38], schools should offer such programmes for teachers.

On the one hand, the results of UTAUT studies among mathematics teachers are ambiguous. On the other hand, there is a complete lack of research investigating the factors that influence these teachers' perceptions of digital tools when applying the UTAUT approach, especially in the one-to-one teaching environment. Hence, the results of our study will provide vital information for mathematics education stakeholders on how to facilitate teachers’ adaptions to DD in a high-potential digital teaching environment.

## 2 Methodological background

### 2.1 Research model and hypotheses

**Fig. 1:**
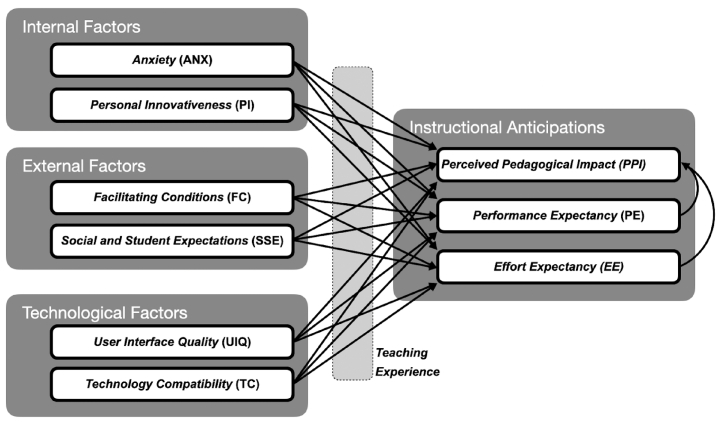
Research model and hypothesis.

This study's hypotheses and research model are based on UTAUT and the model from previous research conducted in similar social environments or that explores acceptance of similar educational technologies [39,40]. The model also considers the legal regulations of Austria that instruct teachers to use DD in teaching processes (see regulations concerning didactic principles https://www.ris.bka.gv.at/GeltendeFassung.wxe?Abfrage=Bundesnormen&Gesetzesnummer=10008568). Furthermore, the mathematics curriculum requires the use of digital technologies (notebooks or graphing calculators) and software (spreadsheet programs or dynamic geometry software) from the beginning of secondary education to explore and discover mathematics, perform calculations and visualise data and mathematical artefacts. The aim of our study is to investigate the factors influencing teachers' instructional anticipations of DD in teaching (see Fig. 1). The study will analyse three aspects of instructional anticipations: Perception of Pedagogical Impact (PPI), Performance Expectancy (PE) and Effort Expectancy (EE). This creates a basis for understanding teachers' perception of DD and the factors that influence its implementation in the educational process, which is crucial for raising the quality of teaching in a digital society.

As it can be expected that there will be more and more digital one-to-one initiatives in schools and that more and more curricula will require the mandatory use of digital technologies, an adaptation of the UTAUT will be necessary. Thus, our study aims, on the one hand, to investigate mathematics teachers’ expectations and anticipations towards technology use and, on the other hand, to propose and utilise a modified model of the UTAUT.

The voluntary use of DD most often indicates that teachers have a positive belief that integrating specific digital tools will improve pedagogical methods in the classroom [41,42]. Research emphasizes that **Perception of Pedagogical Impact (PPI)** is a crucial factor that shapes teachers’ decisions about the acceptance and use of various technological resources in education, such as educational software [43], interactive whiteboards [40] and 3D printers [44]. The research mentioned above indicates that teachers who develop a positive PPI tend to be more inclined to integrate DD into their teaching process because they think that DD enhance students' learning. In our research, PPI is the first dependent outcome construct for which external, internal, and technological factors will be explored.

The second dependent outcome construct is **Performance Expectancy (PE**). The user's belief that using a particular technology will help improve his or her performance is defined as PE [23]. Holzmann et al. [39] point out that PE significantly influences Austrian teachers' perceptions about using 3D printers as an educational tool in the classroom. Other researchers have reached similar conclusions in other social settings and with other technologies. For example, PE is a strong predictor of maths teachers introducing micro-lectures [22] or using dynamic software in the classroom [45].

As a third dependent outcome construct in our research, **Effort Expectancy (EE**) is defined. The user's perception of how easy it is to use digital technology is defined as EE [23]. Holzmann et al. [39] emphasise that EE does not influence the decision of secondary school teachers in Austria to use 3D printers in the classroom. On the other hand, research by authors [46] shows that EE influences teachers' decisions to use mobile technologies in the classroom. Similarly, authors [22] and Venter [45] indicate that EE affects mathematics teachers' decision to implement micro-lectures and mobile technology in teaching. In our research, external, internal, and technological factors that affect PPI, PE, and EE will be explored. Furthermore, we are investigating the extent to which PE and EE influence PPI.

According to UTAUT, teachers’ expectations (PPI, PE, EE) are affected by Anxiety (ANX), Personal Innovativeness (PI), User Interface Quality (UIQ), Technology Compatibility (TC), Social influence and Student Expectations (SSE), and Facilitating conditions (FC).

**Anxiety (ANX)** is understood as the teachers’ opinion that they are afraid of using DD, that they can make a mistake and lose data when using DD in classrooms, and that DD irritate them [39,47]. ANX can negatively affect PE and EE when they apply some technology in class [47,48]. Research by Holzmann et al. [39] indicates that teachers in Austria with a low level of ANX showed higher behavioural intentions in using 3D printers in teaching. Based on previous similar research [39,47], the following hypotheses were set.

H1 ANX has a statistically significant effect on PPI.

H2 ANX has a statistically significant effect on PE.

H3 ANX has a statistically significant effect on EE.

**Personal Innovativeness (PI)** has been defined as a personality attribute that demonstrates a person's excitement or openness to embracing and investigating new technology [49,50]. Previous research indicates that PI affects teachers' PE, PPI, and EE when they adopt new digital technologies in teaching [46,51,52]. In light of this, the following hypotheses were established.

H4 PI has a statistically significant effect on PPI.

H5 PI has a statistically significant effect on PE.

H6 PI has a statistically significant effect on EE.

**User Interface Quality (UIQ)** was defined in previous studies [42,53] as the degree of teacher opinion indicating good organisation of the educational software and ease of navigation through the digital tools. UIQ can significantly influence teachers’ perceptions of the usefulness of educational software in the classroom as well as PPI, EE and PE, and the final decision on whether to use a particular technology in their teaching [42,44,54]. With this in mind, the following hypotheses are proposed.

H7 UIQ has a statistically significant effect on PPI.

H8 UIQ has a statistically significant effect on PE.

H9 UIQ has a statistically significant effect on EE.

**Technology Compatibility (TC)** represents the degree to which the new technology or software is compatible with the existing technology, hardware and software teachers already use in the classroom [55]. Pagani [56] and Šumak et al. [42] suggest that technology compatibility (TC) can significantly influence the adoption of digital technologies in education as well as teachers’ perceptions of PPI, PE and EE that this technology entails. Therefore, the following is hypothesized.

H10 TC has a statistically significant effect on PPI.

H11 TC has a statistically significant effect on PE.

H12 TC has a statistically significant effect on EE.

**Social influence and Student Expectations (SSE)** describe how much individuals take into account the ideas and opinions of others when making decisions about adopting a certain technical solution [23]. Research by Holzmann et al. [39] shows that SSE has an impact on Austrian teachers' behavioural intention to use 3D printers in the classroom. Research in other social settings also suggests that SSE correlates with teachers’ anticipations towards PPI, PE, and EE [43,46]. Based on this, the following hypotheses were set.

H13 SSE has a statistically significant effect on PPI.

H14 SSE has a statistically significant effect on PE.

H15 SSE has a statistically significant effect on EE.

**Facilitating conditions (**FC**)** represent the user's belief that they will receive the necessary support from the organisation they work for in the form of technical equipment and assistance in implementing a particular technology [23]. Holzmann et al.’s [39] study, undertaken in Austria FC, represents one of the factors that most influence the teacher's decision to use 3D printers in the classroom. Authors [46], Pagani [56] and Šumak et al. [42] also indicate that FC can influence PPI, PE and EE. Taking this into consideration, the following hypotheses are proposed.

H16 FC has a statistically significant effect on PPI.

H17 FC has a statistically significant effect on PE.

H18 FC has a statistically significant effect on EE.

In almost all of the above studies [42,43,46,56], years of teaching experience influence the strength of the association between the constructs in UTAUT the model. This was the basis for the final hypothesis in this study.

H19 Years of teaching experience have influenced the degree to which ANX, PI, UIQ, TC, FC and SSE influence PPI, PE, and EE.

### 2.2 Participants and procedure

The teachers relevant to our study are secondary mathematics teachers who taught a fifth or sixth grade in the academic year 2021/2022, as these classes were included in the digital one-to-one initiative. According to STATISTIK AUSTRIA [57], there were 15,361 lower secondary classes (grades 5 to 8; 10 to 14-year-old students) in the academic year 2021/2022. Hence, approximately 7680 classes (grades 5 and 6) and their teachers were relevant to our study. Of these teachers, 69 % were female and 31 % male. Further information on the gender and age distribution of lower secondary school teachers can be found in Table 1.

**Table 1:** Demographic information on the mathematics teachers who participated in our study.

	All data	Fully completed	
Female in our study:	674 (60 %)	569 (62 %)	
Male in our study:	383 (34 %)	321 (35 %)	
No answer:	71 (6 %)	27 (3 %)	
	All data	Fully completed	
1–5 years	196 (17 %)	170 (19 %)	
6–10 years	186 (16 %)	154 (17 %)	
11–15 years	155 (14 %)	128 (14 %)	
16–20 years	113 (10 %)	89 (10 %)	
>20 years	425 (38 %)	364 (40 %)	
No answer	54 (5 %)	13 (1 %)	
	Female	Male	All
<40 years	34 %	33 %	34 %
40–55years	39 %	41 %	40 %
>55 years	27 %	26 %	26 %

**Table 2:** Descriptive statistics and latent correlations of the study variables.

Variable	*N*	#Items	Mean	SD	Alpha	Omega	PPI	PE	EE	ANX	PI	UIQ	TC	SSE	FC
PPI	910	5	4.36	1.39	0.91	0.94	1.00								
PE	910	3	4.90	1.62	0.92	0.92	0.79	1.00							
EE	910	3	5.77	1.31	0.90	0.90	0.42	0.45	1.00						
ANX	910	4	1.36	0.88	0.87	0.89	−0.18	−0.26	−0.52	1.00					
PI	910	4	5.21	1.41	0.89	0.90	0.51	0.52	0.65	−0.37	1.00				
UIQ	910	3	5.41	1.34	0.85	0.86	0.70	0.78	0.48	−0.32	0.51	1.00			
TC	910	3	4.86	1.47	0.82	0.83	0.68	0.80	0.61	−0.36	0.71	0.73	1.00		
SSE	910	3	4.35	1.66	0.84	0.85	0.57	0.49	0.25	−0.09	0.43	0.50	0.52	1.00	
FC	910	3	5.37	1.24	0.69	0.70	0.45	0.54	0.85	−0.53	0.71	0.59	0.73	0.40	1.00

npar	χ^2^	*df*	*p*	CFI	TLI	RMSEA	ci.low	ci.upp	SRMR
129.00	1279.005	398	0.000	0.939	0.929	0.049	0.047	0.052	0.051

In order to generate a high number of participating teachers, we contacted all principals of lower secondary schools and asked them to forward the invitation to participate in our study to the mathematics teachers of the respective schools who teach 5th or 6th grades (see Table 2). Before contacting the school principals, we received written consent from all education directorates in the respective federal states to conduct our study. Participation in our study was voluntary for all teachers as it is required by the ethical board of the University of and educational directorates of Austria. In the invitation to participate in our study, the education directorates, the principals of the schools and the mathematics teachers were informed about the aim of our study and the particular characteristics of the UTAUT. All participants gave written informed consent to participate in the study, which was an integral part of the data collection survey. To ensure the participation of teachers who have different views (positive, neutral and negative) about digital devices, no data was collected that could indicate the teacher's location, the school where they teach, or any information that can indicate their identity. This approach of recruiting participants with different views of digital technologies has been used in previous studies [e.g., 37, 39, 40]. A total of 1263 mathematics teachers accessed our digital questionnaire, 1129 started the questionnaire, i.e., provided demographic information and answered at least five questions, and 918 completed it. Therefore, at least 12 % of the relevant teachers participated in our study. The actual response rate might be higher, as it can be assumed that some mathematics teachers teach more than one grade 5 or 6 class, thus reducing the total population of 7680 teachers relevant to our study. Detailed demographic information on the mathematics teachers who participated in our study can be found in Table 1.

### 2.3 Measures

To cover all categories and their manifestations regarding mathematics teachers’ acceptance and usage of digital technologies, we used nine constructs steaming from other UTAUT studies. The items of the scales were translated into German where necessary and adapted to fit the teaching of mathematics at the lower secondary level context. We developed an online questionnaire using these nine constructs with a total of 40 items, each measured on a 7-point Likert scale (1 = “do not agree,” 7 = “strongly agree”). In order to decrease the likelihood that the teachers use the centre of the seven values as an avoidance option, we also provided them the option “do not want to answer” for each item. The items used in the data collection instrument and the literature on which they are based are listed in Appendix A.

### 2.4 Analytical approach

All models were estimated using the R package ‘MplusAutomation’ [58] in combination with Mplus 8 [59].

To test our hypotheses, a structural equation modeling (SEM) approach was employed. In the first step (H1-H18), we tested a mediation model with the internal, external, and technological factors as predictors, teachers’ PPI as the outcome and PE and EE as mediators. We used bootstrapping (1000 samples) to estimate the standard errors and confidence intervals for the indirect effects (see Table 4) of the internal, external, and technological factors on the perception of pedagogical impact and via performance expectancy and effort expectancy [60]. In a second step (H19), latent variable interaction was examined to understand the moderating effect of teaching experience on the relationship between the internal, external, and technological factors and outcome variables PPI and via PPE and EE. The interaction term was specified using the XWITH command in Mplus 8. This approach accounts for measurement error in the latent variables, providing a more accurate estimation of the interaction effect. The model was estimated using the numerical integration method in combination with random slopes, i.e. allowing the slopes of one interaction variable to vary across the degree of the other interaction variable [61]. In a third step, in addition to latent interaction analysis, we performed an “extreme” group comparison by means of multiple group analysis. More specifically, the hypothesized model was tested for teachers with less than 10 years of teaching experience and for teachers with more than 16 years of teaching experience. Prior to multiple group analysis, we tested the study variables for measurement invariance [62].

In all three steps, the robust maximum likelihood estimator was employed, as this estimator allows for corrections to standard errors when observed data deviate from normality [63].

In the present study, missing values should not pose a particular risk to the validity of the findings, as only two items (SSE) had more than five per cent missing values. Nevertheless, to avoid dropouts due to missing data, we used full-information maximum likelihood estimation, which is an appropriate treatment of missing data under the MAR mechanism [64]. To test the MAR assumption, we performed the Hawkins’ test (median Χ^2^(14) = 432.8224, median *p* < 00.001) which indicates that the missing data was the consequence of a missing at random (MAR; see Ref. [65]) mechanism, i.e., the missingness depends on other study variables.

Due to the lack of information on teachers’ school affiliation, it was not possible to take the sample clustering (teachers nested in schools) into account.

## 3 Results

### 3.1 Descriptive statistics

In the results section of our study, we begin by presenting the descriptive statistics (see Table 2), which reveal several notable findings. Firstly, all latent correlations (χ^2^ = 1279.005, df = 398, χ^2^/df = 3.215, CFI = 0.939, TLI = 0.929, RMSEA = 0.049 [0.047, 0.052], SRMR = 0.051) identified in our analysis are statistically significant, with *p*-values less than 0.001, indicating a strong likelihood that these relationships are not due to chance. An exception to this pattern is the correlation between ANX and SSE, which, while significant, presents a p-value of 00.027, suggesting a less robust but still existing relationship.

Regarding the predictors within our study, some exhibit high correlations among themselves, with correlation coefficients exceeding 0.7. Despite these high correlations, they do not reach a threshold that would indicate concerns about multicollinearity, suggesting that each predictor contributes unique information to our model. Particularly notable is the correlation between PPI and PE, which stands at 0.79. However, the shared variance (R^2^) between these two constructs is 62 %, indicating that while they are closely related, they are empirically distinct constructs.

We also observed significant associations between specific predictors, namely UIQ and TC, and the dependent variables PPI and PE. Similarly, FC and EE demonstrate a strong relationship. These relationships underscore the potential influence of these predictors on the outcomes of interest.

In terms of central tendency and dispersion, mean values and standard deviations across variables do not present any unusual patterns. Notably, ANX is characterized by very low mean values, whereas EE, PI, UIQ, and FC exhibit higher mean values, suggesting a tendency towards higher expressions of these constructs in our sample.

Lastly, the reliability coefficients for the scales used in our study are consistently high, indicating high measurement precision (McDonald's ω: PE = 0.924; EE = 0.907; PPI = 0.912; PION = 0.888; ANX = 0.879; UIQ = 0.867; TC = 0.827; SSE = 0.842; FC = 0.783). This reliability underscores the robustness of our findings and the reliability of the instruments employed to assess the constructs of interest in our research.

These findings lay the groundwork for further analyses and discussions regarding the implications of these relationships and the role of the identified predictors in influencing the dependent variables within the context of our study.

### 3.2 Step 1: testing hypotheses on direct and indirect effects of internal, external, and technological factors on perceived pedagogical impact

#### 3.2.1 Direct Effects

The overall fit of our hypothesized model (see Fig. 1) was good (χ^2^ = 1576.757, df = 399, χ^2^/df = 3.951, CFI = 0.939, TLI = 0.929, RMSEA = 0.049 [0.047, 0.052], SRMR = 0.051), indicating that our mediation model reasonably accounted for the data. The model also has substantial explanatory power, since significant portions of the variance in the mediators (i.e., 73 % in PE and 75 % in EE) and in the outcome (70 % in PI) were explained. Standardized path coefficients for all direct effects are presented in Table 3.

**Table 3:** Direct effects.

H	lhs	rhs	est	se	*p*	ci.lower	ci.upper	std.all
	PPI	EE	0.239	0.086	0.006	0.070	0.409	0.231
	PPI	PE	0.483	0.059	0.000	0.367	0.600	0.542
1	PPI	ANX	0.076	0.055	0.169	−0.032	0.184	0.047
2	PE	ANX	0.037	0.057	0.512	−0.074	0.149	0.021
3	EE	ANX	−0.133	0.084	0.114	−0.298	0.032	−0.085
4	PPI	PI	0.097	0.044	0.028	0.011	0.184	0.114
5	PE	PI	−0.050	0.050	0.320	−0.147	0.048	−0.052
6	EE	PI	0.108	0.058	0.061	−0.005	0.221	0.131
7	PPI	UIQ	0.176	0.052	0.001	0.074	0.278	0.178
8	PE	UIQ	0.473	0.063	0.000	0.349	0.598	0.426
9	EE	UIQ	0.008	0.091	0.929	−0.170	0.186	0.008
10	PPI	TC	0.018	0.081	0.823	−0.142	0.178	0.018
11	PE	TC	0.647	0.083	0.000	0.483	0.810	0.575
12	EE	TC	−0.017	0.096	0.862	−0.206	0.172	−0.017
13	PPI	SSE	0.170	0.030	0.000	0.111	0.229	0.224
14	PE	SSE	0.039	0.030	0.187	−0.019	0.098	0.046
15	EE	SSE	−0.083	0.031	0.008	−0.145	−0.022	−0.114
16	PPI	FC	−0.355	0.146	0.015	−0.642	−0.069	−0.309
17	PE	FC	−0.119	0.081	0.141	−0.277	0.039	−0.092
18	EE	FC	0.851	0.128	0.000	0.601	1.101	0.766

npar	χ^2^	*df*	*p*	CFI	TLI	RMSEA	ci.low	ci.upp	SRMR
128.00	1576.757	399	0.000	0.939	0.929	0.049	0.047	0.052	0.051

**Table 4:** Total, total indirect and direct effects.

pred	outcome	summary	low.5	low2.5	low5	est	up5	up2.5	up.5
ANX	PPI	Total	−0.068	−0.049	−0.044	0.036	0.090	0.100	0.132
ANX	PPI	Total indirect	−0.093	−0.093	−0.071	−0.011	0.012	0.017	0.024
ANX	PPI	Direct	−0.031	−0.012	−0.004	0.047	0.097	0.118	0.122
PI	PPI	Total	−0.045	−0.002	0.021	0.119	0.243	0.261	0.270
PI	PPI	Total indirect	−0.095	−0.071	−0.038	0.007	0.065	0.068	0.089
PI	PPI	Direct	−0.035	−0.003	0.025	0.113	0.218	0.228	0.255
UIQ	PPI	Total	0.284	0.301	0.333	0.411	0.513	0.532	0.536
UIQ	PPI	Total indirect	0.076	0.127	0.142	0.229	0.329	0.329	0.351
UIQ	PPI	Direct	0.038	0.038	0.084	0.182	0.273	0.288	0.304
TC	PPI	Total	0.126	0.146	0.159	0.329	0.422	0.439	0.477
TC	PPI	Total indirect	0.129	0.182	0.208	0.302	0.411	0.428	0.449
TC	PPI	Direct	−0.165	−0.165	−0.155	0.027	0.111	0.142	0.185
SSE	PPI	Total	0.129	0.129	0.133	0.223	0.274	0.283	0.295
SSE	PPI	Total indirect	−0.119	−0.051	−0.038	−0.001	0.042	0.056	0.070
SSE	PPI	Direct	0.139	0.139	0.139	0.224	0.269	0.280	0.297
FC	PPI	Total	−0.437	−0.349	−0.327	−0.192	−0.098	−0.066	−0.042
FC	PPI	Total indirect	−0.015	−0.015	−0.006	0.118	0.286	0.335	0.395
FC	PPI	Direct	−1.131	−0.630	−0.587	−0.310	−0.140	−0.124	−0.097

H1, H2, H3 stated that ANX is related to the three outcome dimensions (PPI, PE, EE), however, the results indicated that ANX did not significantly predict any of the outcome dimensions (see Table 4). Thus, H1, H2, H3 were not supported.

H4, H5, H6 posited a significant relationship between PI and the three outcome dimensions. However, analyses revealed that PI was only significantly related to PPI (β = 0.114, p = 0.028) but not to PE and EE. Thus, while H4 was supported, H5 and H6 were rejected.

H7, H8, H9 stated that UIQ is related to the three outcome dimensions. While UIQ significantly predicted PPI (β = 0.178, p = 0.001) and PE (β = 0.426, p < 0.001), UIQ was not significantly related to EE. Thus, H7 and H8 were supported; H9 was rejected.

H10, H11, H12 posited a significant relationship between TC and the three outcome dimensions, however, the results indicated that only PE was significantly predicted by TC (β = 0.575, p < 0.001). Thus, only H 11 was supported.

H13, H14, H15 stated that SSE is related to the three outcome dimensions, however, the results indicated this was only true for PPI (β = 0.224, p < 0.001), whereas SSE had a significant negative relation with EE (β = −0.114, p = 0.008) and an insignificant one with PE. Thus, only H13 was supported.

H16, H17, H18 posited a significant relationship between FC and the three outcome dimensions. Indeed, FC showed a strong association with EE (β = 0.766, p < 0.001) but was significantly negatively associated with PPI (β = −0.309, p = 0.015) and showed no significant relation with PE. Thus, only H18 was supported.

#### 3.2.2 Indirect effects

Table 5, Table 6 report the results of indirect effects testing. As can be seen from the bias-corrected confidence intervals of the specific indirect effects table (Table 5), UIQ, TC, SSE, and FC exerted indirect effects via PE or EE on PPI. Particularly strong were the indirect effects of UIQ and TC via PE on PPI (β = 0.227 [0.151, 0.310]; β = 0.310 [0.230, 0.428]) (see Table 7).

**Table 5:** Specific indirect effects.

pred	intervening	outcome	low.5	low2.5	low5	est	up5	up2.5	up.5
ANX	PE	PPI	−0.044	−0.044	−0.037	0.008	0.031	0.035	0.043
ANX	EE	PPI	−0.100	−0.100	−0.048	−0.019	−0.002	0.003	0.010
PI	PE	PPI	−0.106	−0.080	−0.071	−0.024	0.021	0.029	0.044
PI	EE	PPI	−0.043	−0.025	−0.003	0.031	0.069	0.071	0.089
UIQ	PE	PPI	0.120	0.151	0.156	0.227	0.286	0.310	0.337
UIQ	EE	PPI	−0.053	−0.040	−0.030	0.002	0.031	0.043	0.064
TC	PE	PPI	0.175	0.230	0.243	0.310	0.424	0.428	0.441
TC	EE	PPI	−0.130	−0.077	−0.061	−0.007	0.036	0.040	0.045
SSE	PE	PPI	−0.015	−0.005	−0.002	0.025	0.060	0.066	0.083
SSE	EE	PPI	−0.138	−0.067	−0.061	−0.026	−0.010	−0.006	−0.002
FC	PE	PPI	−0.130	−0.122	−0.119	−0.060	−0.013	−0.003	0.017
FC	EE	PPI	0.042	0.042	0.053	0.179	0.370	0.465	0.505

**Table 6:** Latent interaction effects.

Model	*N*	Par	AIC	BIC	aBIC	AICC	std est	p value
EE|ANX	902	131.00	82546.495	83175.899	82759.864	82591.409	−0.051	0.243
EE|FC	902	131.00	82534.359	83163.763	82747.729	82579.273	0.098	0.001
EE|PI	902	131.00	82547.111	83176.515	82760.480	82592.025	0.048	0.085
EE|SSE	902	131.00	82550.931	83180.336	82764.301	82595.845	−0.009	0.748
EE|TC	902	131.00	82546.268	83175.673	82759.638	82591.182	0.053	0.071
EE|UIQ	902	131.00	82537.663	83167.068	82751.033	82582.577	0.084	0.016
PE|ANX	902	131.00	82588.639	83218.043	82802.008	82633.553	−0.049	0.029
PE|FC	902	131.00	82592.097	83221.502	82805.467	82637.011	0.023	0.332
PE|PI	902	131.00	82592.957	83222.361	82806.326	82637.871	0.000	0.986
PE|SSE	902	131.00	82592.956	83222.361	82806.326	82637.870	0.002	0.944
PE|TC	902	131.00	82592.832	83222.237	82806.202	82637.746	0.008	0.678
PE|UIQ	902	131.00	82591.989	83221.393	82805.358	82636.903	0.022	0.309
PPI|ANX	902	131.00	82586.458	83215.862	82799.827	82631.372	−0.025	0.325
PPI|FC	902	131.00	82586.907	83216.311	82800.277	82631.821	0.020	0.394
PPI|PI	902	131.00	82586.959	83216.363	82800.328	82631.873	0.019	0.427
PPI|SSE	902	131.00	82585.904	83215.308	82799.273	82630.818	0.031	0.203
PPI|TC	902	131.00	82583.161	83212.565	82796.530	82628.075	0.051	0.027
PPI|UIQ	902	131.00	82584.143	83213.548	82797.513	82629.057	0.043	0.047

**Table 7:** Multigroup analysis.

DV	IV	Novice_est	Novice_se	Novice_p	Expert_est	Expert_se	Expert_p	Delta_est	Delta_es	Delta_p
PPI	ANX	0.058	0.059	0.324	0.004	0.057	0.943	0.054	0.082	0.508
PPI	PI	0.112	0.063	0.075	0.159	0.108	0.141	−0.047	0.124	0.708
PPI	UIQ	0.292	0.107	0.006	0.039	0.101	0.701	0.253	0.147	0.085
PPI	TC	−0.102	0.131	0.439	0.241	0.212	0.256	−0.342	0.249	0.170
PPI	SSE	0.213	0.060	0.000	0.275	0.092	0.003	−0.062	0.110	0.572
PPI	FC	−0.348	0.257	0.176	−1.147	0.732	0.117	0.799	0.773	0.301
EE	ANX	−0.064	0.167	0.701	−0.014	0.092	0.876	−0.050	0.191	0.795
EE	PI	0.158	0.105	0.131	−0.081	0.099	0.413	0.239	0.144	0.096
EE	UIQ	−0.220	0.101	0.029	0.225	0.134	0.094	−0.445	0.168	0.008
EE	TC	0.142	0.152	0.350	−0.283	0.180	0.115	0.425	0.231	0.066
EE	SSE	−0.024	0.065	0.711	−0.191	0.063	0.002	0.167	0.090	0.063
EE	FC	1.096	0.268	0.000	1.638	0.287	0.000	−0.542	0.389	0.163
PE	ANX	0.090	0.063	0.150	−0.018	0.055	0.745	0.108	0.083	0.193
PE	PI	−0.001	0.072	0.986	−0.026	0.079	0.747	0.024	0.107	0.821
PE	UIQ	0.496	0.088	0.000	0.471	0.083	0.000	0.025	0.120	0.837
PE	TC	0.716	0.132	0.000	0.595	0.120	0.000	0.121	0.178	0.497
PE	SSE	0.065	0.058	0.258	0.021	0.048	0.666	0.044	0.075	0.555
PE	FC	−0.311	0.143	0.030	−0.126	0.160	0.432	−0.185	0.213	0.384

npar	χ^2^	*df*	*p*	CFI	TLI	RMSEA	ci.low	ci.upp	SRMR
214.00	1638.429	840	0.000	0.934	0.927	0.050	0.046	0.053	0.057

### 3.3 Step 2: testing the moderating effect of teaching experience by means of latent interaction analysis

In our subsequent analysis, we explored the moderating effects within our model using latent interaction effects. This approach aimed to uncover how teaching experience might alter the strength or direction of relationships between our primary predictors and outcomes. The findings reported in Table 6 indicated that significant moderating effects were present in only five instances out of 18. It appears that the relationship between TC, UIQ, and PPI was influenced by age, with a slight increase as age progresses (β = 0.051, *p* = 0.027; β = 0.043, *p* = 0.047). The same applied to the relationship between FC, UIQ, and EE (β = 0.098, *p* = 0.001; β = 0.084, *p* = 0.016). Additionally, the negative impact of ANX on PE seemed to intensify with age (β = −0.049, *p* = 0.029). However, it is important to note that the effect sizes associated with these significant interactions are quite small. This raises questions about the practical significance of these moderating effects and whether they warrant substantive interpretation.

### 3.4 Step 3: testing the moderating effect of teaching experience by means of multiple group analysis

We begin by addressing the findings of our measurement invariance testing before presenting the findings from the Multiple Group Analysis (see Table 7). The results (see Table A1 in the Appendix) reveal that all scales demonstrate both configural and metric invariance, allowing us to compare regression coefficients between the two groups, namely novices and experts. The subsequent multiple group analysis (χ^2^ = 1638.429, df = 840, χ^2^/df = 1.951, CFI = 0.934, TLI = 0.927, RMSEA = 0.050 [0.046, 0.053], SRMR = 0.057) reveals that only the regression coefficient of UIQ on PE is significantly stronger among experts compared to novices (Delta β = 0.168, *p* = 0.008). The effects of PC on EE and SSE on EE are also more pronounced among experts than novices, albeit narrowly missing statistical significance. This finding from the present “extreme group” comparison confirms the insight gained in step 2, indicating that the influence of UIQ on EE varies with the degree of teaching experience.

## 4 Discussion

Relying on the UTAUT, this study investigates the factors that influence the expectations and anticipations of mathematics teachers towards the use of DD in teaching in an educational environment where every student has his or her own DD. Our study has contributed to three main areas in the existing body of knowledge, which are reflected in the description of the correlation between the constructs in the model, the indirect influence in the model, as well as the modelling of the moderating effect of teachers’ experience. These contributions are discussed in detail below.

**Correlation between constructs.** Results of our study show that there is a strong correlation between certain constructs, for example, ANX and SSE. Based on a detailed analysis of previous similar research [39,40,44], a similar correlation was not found. The results of our study indicate that if teachers believe that their social environment expects them to use digital technologies, their anxiety about the use of technologies will increase. Our findings are also supported by the results of Ertmer [41], which indicate that when teachers feel pressure or expectations to incorporate technologies into their teaching practices, they may experience increased anxiety. These results are particularly significant, indicating the importance of careful preparation of teachers for the application of digital technologies in teaching. Further, the results of our study indicate that PPI and PE are two different constructs but have a very significant mutual correlation. Our results provide additional support to the previous results presented by Šumak and Šorgo [40] and authors [44], which indicated a correlation between these two constructs. Also, the results of this study indicate significant correlations between UIQ and TC. We were not able to find a similar correlation in previous research. Nevertheless [66,67], indicate that there is a correlation in teachers' opinions between the displayed teaching content and the quality of the technology used to display it. These data can be of particular importance for the creators of educational policies because they indicate the importance of aligning educational technologies in schools. The findings from our research also indicate a strong correlation between FC and EE. The conclusions drawn from our investigation provide additional support to studies by authors [44], in which the correlation between FC and EE was indicated. These results indicate the importance of supporting teachers when employing technologies in teaching.

**Direct Effects between constructs**: Contrary to expectations (H1, H2, H3), ANX did not significantly predict any of the parameters examined (PPI, PE, EE), suggesting that teachers' fear or discomfort with technology use may not be a significant factor in their perceived impact, performance expectations, or effort expectations. These results contrast with similar previous ones carried out by Holzmann et al. [39], which indicate that the anxiety of Austrian teachers significantly affects their perceptions of the use of 3D printers in teaching. It is possible that the higher demands and complexity of using 3D printers in relation to digital tools offered in Austrian education in the environment of one digital device for each student is the reason for such results. These results indicate the importance of our study and the contribution to knowledge related to the specifics of technology selection in educational environments where each student is equipped with his or her device. On the other hand, personal innovativeness PI (H4) significantly influenced PPI, PE, and EE, in line with the existing literature [40], which emphasizes the role of individual openness and innovativeness towards the adoption of new technologies. These results indicate that teachers with more personal innovation are more likely to accept digital technologies in teaching and more positively evaluate their contribution to teaching. Our investigation reveals that UIQ positively influenced PPI and PE (H7, H8), highlighting the importance of well-organized and transparent educational software in shaping teachers' perceptions and expectations. Our research mirrors the results of authors [44], who indicate that a well-organized user interface directly affects teachers' anticipations about the pedagogical value of a technology and its effectiveness. These data are of great importance to policymakers because they indicate that UIQ is one of the main factors to be taken into consideration when choosing digital tools for schools. According to our results, technology compatibility (TC) significantly predicted performance expectancy (PE) (H11), highlighting the importance of aligning new technologies with existing classroom tools and practices. This provides additional support to the results of previous studies by Inan and Lowther [68] and Francom and Moon [69], which indicate the importance of harmonizing new technologies with existing ones in teaching to improve the expected performance. Surprisingly, the results of our study indicate that teachers believe that TC does not affect PPI or EE. One of the possible explanations is that the teachers probably think that before utilizing technologies in the classroom, all technologies should be assembled and then used in classes. Besides our results, this assumption is based on results of previous research by Ertmer et al. [70] and Maniva et al. [71], which pointed out that teachers' perceptions are more based on their pedagogical contribution in the classroom than their pure technological characteristics. This leads to the conclusion that the harmonization of technologies in classrooms would not significantly affect teachers’ PPI and EE. Future research should investigate these hypotheses. According to findings from our research, social influence and student expectations (SSE) had a significant positive impact on perceived pedagogical impact (PPI) (H13) but a negative impact on effort expectancy (EE). This indicates that the teachers consider the extent to which the technologies that the students expect to be used are used in the classroom; this can improve the effectiveness of the classroom, but certainly not the ease of using the technologies in the classroom. This assumption, in addition to outcomes obtained through in our study, is also supported by the results of 10.13039/100006520Edwards [72] and Dolenc et al. [73], who indicate that the alignment of educational technologies with students' expectations improves their effectiveness in teaching. The results of our study indicate that FC showed a strong positive relationship with EE, PI, and PPI, highlighting the key role of institutional support, technical infrastructure, and assistance in shaping teachers' expectations regarding the effort required for technology integration. Similar results were shown in many previous studies [68,69], which indicate the importance of supporting teachers in the integration of new technologies and new approaches in teaching.

**Indirect Influences: Uncovering Pathways through Mediators.** The results of our study indicate that user interface quality (UIQ) and technology compatibility (TC) had significant indirect effects on perceived pedagogical impact (PPI) through performance expectations (PE). These results highlight the importance of well-designed interfaces and their alignment with existing technology in schools since they can influence teachers’ perception of the pedagogical value of a specific technology. These results are very significant because they contribute to the general body of knowledge in technology acceptance since we could not find similar effects between those above-mentioned constructs in any research using UTAUT as the research background. However, the results of Dikmen and Demirer [74] and Al-Seghayer [75] obtained by employing other research approaches, such as Technological Pedagogical Content Knowledge (TPACK), indicate that the user interface can have an indirect effect on teachers' perceptions of educational technologies. This indicates complex pathways through which certain characteristics of technology influence teachers' perceptions of technology that should be examined in detail in future research.

**The moderating effect of teaching experience: Uncovering contextual dynamics.** Teaching experience was investigated as a potential moderator, influencing the relationships between internal, external, and technological factors and the outcome variables. Although the latent interaction analysis indicated significant moderating effects in five cases, the effect sizes were small, raising questions about their practical significance. In previous research [40,46], the influence of teachers' years of experience on their perceptions of technology was not indicated. Furthermore, the results of our research indicate that only the regression coefficient of UIQ on PE is significantly stronger among more experienced teachers compared to beginners. These results were not found in the available previous research. These results point to two assumptions: 1) that for more experienced teachers quality of the interface that digital devices have is more important than for younger teachers, and 2) that teachers in the initial stages of their careers quickly adapt to different qualities of the interface. Future research should examine these hypotheses. In addition, the results suggest that the effects of PE on EE and SSE on EE are slightly more pronounced among expert teachers, suggesting intriguing trends. Although they did not reach conventional significance levels, these trends indicate a potential pattern in which the influence of pedagogical content and self-confidence on engagement may increase with increasing expertise. Although further research may be needed to determine the robustness of these trends, they nevertheless provide valuable insights into the complex interplay between various factors that shape educational experiences.

We are aware of the potential limitations of our study. Firstly, the main drawback of the study is that the law in Austria strongly recommends the use of digital devices in a one-to-one teaching environment. These recommendations could potentially influence the concepts presented by the teachers. Secondly, the one-to-one approach is a new educational strategy in Austria, so this study does not provide insight into the opinions of teachers who have been working in such a pedagogical setting for a long time.

Our results may have substantial implications for educational policymakers, especially in the context of one-to-one educational environments. As the results of our study indicate, UIQ has proven to be a critical factor that can influence teachers' perceptions of the use of DD in teaching. When equipping students with DD, it is essential that tools with a suitable interface for teaching and learning are selected. In addition, our findings suggest that policymakers and school leaders should consider the technology already present in schools when equipping students with digital devices. The compatibility of DD used by students in one student one digital device in the educational environment with other digital equipment (projectors, computers, printers etc) in schools would improve the digitization of the teaching process. Given that teachers with higher levels of personal innovativeness (PI) are more likely to adopt digital tools positively, teacher training creators should promote examples of good practice and encourage teachers to be innovative. Experienced teachers showed a stronger relationship between UIQ and PE. These data can be of particular importance to school managers. Developing school strategies that connect experienced teachers with younger colleagues through a collaborative mentoring relationship could help overcome these differences by fostering a collaborative and supportive teaching environment.

## 5 Conclusion

This study provides a comprehensive analysis of mathematics teachers' anticipations toward technology integration in a one-student-one-digital-tool educational environment. As far as we know, this is one of the rare studies that explores mathematics teachers' conceptions of using digital devices in a one-to-one teaching environment, using UTAUT as a research background. Considering that almost all previous research in this area emphasized that PPI, PE, and EE are the main constructs that influence the decision of teachers to use certain technologies in teaching, this research took these constructs as output constructs. Lower correlation between ANX and SSE, PPI and PE, UIQ and TC, PPI and PE, FC, and EE in mathematics teachers who teach in a one-on-one educational environment. Significantly mathematics teachers ANX is positively related to the PPI, PE, and EE but does not significantly predict them. Descriptive statistics indicate significant latent correlations, where all identified correlations are statistically significant, except between ANX and the influence of SSE, which is less robust. Associations between certain predictors and dependent variables are also significant, emphasizing their potential influence on the outcomes of interest, such as PPI and PE, FC and EE, and between UIQ and PPI and PE. The results of our study indicate a relationship between UIQ, PPI, and PE. A positive association of mathematics teachers' anticipations of UIQ with PPI and PE, suggests that a high-quality interface positively affects the perception of the pedagogical process and students' expectations of their performance. On the other hand, some hypotheses were not supported. For example, the direct relationship between ANX and outcome dimensions (PPI, PE, EE) was not statistically significant, meaning that anxiety did not have a direct effect on these dimensions of perceived pedagogical impact. In addition, the results indicate that UIQ and TC have indirect effects through PE on the PPI. These indirect effects indicate that factors can influence PPI through mediating factors, which provides additional complexity in understanding these relationships. Our results also indicate that the impact of user interface quality on performance expectancy is stronger among expert teachers compared to novices. The results obtained in our study can be very useful for policymakers and. school administrators because they indicate important information that should be paid attention to when creating a digital one-to-one teaching environment. In addition, our work provides hypotheses for future research that should be investigated.

## Funding

This research did not receive any specific grant from funding agencies in the public, commercial, or not-for-profit sectors.Ethics approva

This study was approved by the Ethics Committee of the School of Education, Johannes Kepler University in Linz, with ethics approval reference 379005855786.

## Availability of data and material (data transparency)

All data and materials as well as software application or custom code support published claims and comply with field standards. The data generated during and/or analyzed during the current study are available from the corresponding author on reasonable request.

## Code availability (software application or custom code)

Not applicable.

## Ethics approval (include appropriate approvals or waivers)

All procedures followed were in accordance with the ethical standards and principles of conducting research at the School of Education, Johannes Kepler University in Linz. Furthermore, consensus to conduct our study was obtained from all educational directorates of the federal states in Austria.

## Research involving human participants - rights

The study involved human participants (primary and lower secondary school teachers) who voluntarily chose to participate in the research. All research participants are guaranteed privacy and anonymity.

## Informed consent

For the realization of the research, permission (consent) was sought from primary school principals, school pedagogues and psychologists, as well as teachers themselves. Participation in the study was voluntary. The data was collected, saved and analyzed anonymously.

## Consent for publication

All authors have read and approved the final version of the article.

## CRediT authorship contribution statement

**Robert Weinhandl:** Writing – original draft, Visualization, Validation, Methodology, Investigation, Formal analysis, Data curation, Conceptualization. **Christoph Helm:** Writing – original draft, Visualization, Validation, Supervision, Methodology, Investigation, Formal analysis, Data curation. **Branko Anđić:** Writing – original draft, Methodology, Investigation, Data curation. **Cornelia S. Große:** Writing – original draft, Investigation.

## Declaration of competing interest

The authors declare that they have no conflict of interest.

## Footnotes

1 https://www.grandviewresearch.com/industry-analysis/education-technology-market# (08.02.2024).
