# Supplementary material for: Uncovering mathematics teachers’ instructional anticipations in a digital one-to-one environment: A modified UTAUT study
Source: Heliyon. 2024 Aug 3;10(15):e35381. doi: 10.1016/j.heliyon.2024.e35381 (PMC11336610; doi:10.1016/j.heliyon.2024.e35381)
Supplement: Multimedia component 1 [file mmc1.docx]

| **Appendix A**  Items in the data collection instrument: | Literature on the basis of which the items were developed: |
| --- | --- |
| **Performance Expectancy (PE)** | [36, 42, 43] |
| 1. DDs are useful for my teaching. |  |
| 2. Using DDs increases my productivity in teaching. |  |
| 3. Using DDs improves the quality of my teaching. |  |
| 4. Using DDs makes it easier to conduct my teaching. |  |
| 5. Using DDs enables me to complete tasks related to my teaching more quickly. |  |
| **Effort Expectancy (EE)** | [36, 42] |
| 1. DDs are easy for me to use. |  |
| 2. Acquiring the skills to use DDs in teaching is easy for me. |  |
| 3. How to operate DDs is clear to me. |  |
| 4. It takes too long to learn how to use DDs in my teaching to be worth the effort. |  |
| **Perceived Pedagogical Impact (PPI)** | [37, 40] |
| 1. Using DDs in my teaching has a positive impact on students' critical thinking skills. |  |
| 2. Using DDs in my teaching positively impacts students' active participation. |  |
| 3. Using DDs in my teaching positively impacts students' motivation. |  |
| 4. Using DDs in my teaching positively impacts students' academic performance. |  |
| 5. Using DDs in my teaching positively impacts students' attitudes towards learning. |  |
| 6. Using DDs in my teaching positively impacts the time students spend using the internet and digital resources for learning purposes. |  |
| **User Interface Quality (UIQ)** | [39, 51, 52] |
| 1. It's easy for me to navigate the DG user interface. |  |
| 2. The DG user interface is interactive and well-organized. |  |
| 3. Teaching content is well-presented through the use of DDs. |  |
| 4. Using DDs in teaching creates an audio-visual experience. |  |
| 5. Teaching content can be presented attractively through the use of DDs. |  |
| **Technology Compatibility (TC)** | [39, 53, 54] |
| 1. The DG software is compatible with other devices I use (private & school computers, tablets, PCs, smartphones, etc.). |  |
| 2. Using DDs fits my lifestyle. |  |
| 3. Using DDs is compatible with all aspects of my teaching. |  |
| 4. Using DDs is compatible with the teaching methods I typically use in teaching. |  |
| **Personal Innovativeness (PI)** | [49, 50] |
| 1. I enjoy experimenting with new technologies. |  |
| 2. I enjoy experimenting with new teaching materials. |  |
| 3. Generally, I am not hesitant to try new technologies in teaching. |  |
| 4. When I hear about new technology, I look for ways to experiment with it. |  |
| **Social influence and Student Expectations (SSE)** | [36, 40] |
| 1. Students expect me to use DDs in teaching. |  |
| 2. Students' parents expect me to use DDs in teaching |  |
| 3. People who influence my private and/or school life believe I should use DDs. |  |
| 4. Colleagues at my school who use DDs have more prestige than those who don't. |  |
| **Anxiety (ANX)** | [36, 45, 46] |
| 1. I'm afraid to use DDs in teaching. |  |
| 2. The thought that I could lose a lot of information if I press the wrong button when using DDs scares me. |  |
| 3. I hesitate to use DDs because I'm afraid of making mistakes I can't correct. |  |
| 4. DDs irritate me. |  |
| **Facilitating Conditions (FC)** | [36, 39, 54] |
| 1. I have the necessary resources (time, internet, software, etc.) to use DDs in teaching. |  |
| 2. I have the necessary knowledge and skills to use DDs in teaching. |  |
| 3. DDs are compatible with other resources (textbooks, workbooks, projectors, etc.) that I use in teaching. |  |
| 4. At our school, a specific person (or group) is available to provide support with DG-related difficulties. |  |
